# Effects of Zinc Diethyldithiocarbamate (ZDC) on Rheological Behavior and Aging Resistance of SBS-Modified Asphalt

**DOI:** 10.3390/ma19132893

**Published:** 2026-07-06

**Authors:** Zhenshi Zhong, Shi Xu, Shichao Liang, Xiongjiang Wang, Yongping Hu, Georgios Pipintakos, Shisong Ren, Quantao Liu, Shaopeng Wu

**Affiliations:** 1 School of Civil Engineering and Architecture, Wuhan University of Technology, Wuhan 430070, China; 2 Hubei Key Laboratory of Roadway Bridge and Structure Engineering, Wuhan University of Technology, Wuhan 430070, China; 3 Department of Civil and Environmental Engineering, National University of Singapore, Singapore 117577, Singapore; 4 SuPAR Research Group, University of Antwerp, 2020 Antwerp, Belgium; 5 State Key Laboratory of Silicate Science and Advanced Building Materials, Wuhan University of Technology, Wuhan 430070, China

**Keywords:** SBS-modified asphalt, ZDC, aging resistance, thermo-oxidative and ultraviolet aging, DSR, FTIR

## Abstract

Aging of Styrene–butadiene–styrene (SBS)-modified asphalt accelerates the degradation of both the SBS polymer network and asphalt components, resulting in deterioration of the durability of asphalt concrete. This study investigates the use of zinc diethyldithiocarbamate (ZDC), a multifunctional antioxidant, in SBS-modified asphalt to improve its aging resistance. Physical property tests, dynamic rheological analysis, multiple stress creep recovery (MSCR) and Fourier transform infrared spectroscopy (FTIR) assays were conducted to evaluate the rheological and chemical properties of asphalt binders before and after thermo-oxidative and UV aging. The results indicate that the incorporation of ZDC improved the deformation resistance and elastic recovery of SBS-modified asphalt. After aging, the ZDC/SBS composite-modified asphalt exhibited lower performance change rate than conventional SBS-modified asphalt, indicating enhanced resistance to permanent deformation and aging-induced damage. FTIR analysis demonstrated that ZDC effectively inhibited the formation of oxygen-containing functional groups during aging, suggesting suppressed oxidative reactions within the asphalt binder. The 5% ZDC dosage reduces the carbonyl index of SBS-modified asphalt by 36.48% after thermo-oxidative aging, and by 21.89% after UV aging, showing a stronger chemical inhibition effect on thermo-oxidative reactions. From the perspective of rheological performance stability, ZDC lowers the variation amplitude of non-recoverable creep compliance by 35.32% before and after thermo-oxidative aging and 41.46% before and after UV aging, and delivers a more prominent mitigating effect on property fluctuations triggered by UV aging. This indicates that ZDC exerts differentiated anti-aging mechanisms on thermo-oxidative and UV aging, with considerable potential to improve the comprehensive aging resistance of polymer-modified asphalt binders.

## 1. Introduction

Styrene–butadiene–styrene (SBS)-modified asphalt has been widely used in pavement engineering due to its remarkable resistance to high-temperature rutting, low-temperature cracking and fatigue damage [1]. Through high-shear mixing, SBS can form a relatively stable polymer network structure within the asphalt binder, thereby significantly improving the viscoelastic properties and overall pavement performance of conventional asphalt [2].

Despite its superior performance, SBS-modified asphalt is susceptible to aging during long-term service under the combined effects of heat, oxygen, ultraviolet (UV) radiation and moisture [3,4]. Thermo-oxidative aging can induce oxidation and volatilization of light components in asphalt, while UV radiation further accelerates molecular degradation and polymer chain scission of SBS [5]. These aging processes gradually destroy the polymer network structure and increase the stiffness and brittleness of the binder, resulting in deterioration of rheological properties, reduced fatigue resistance, and premature pavement distress [6,7]. Therefore, enhancing the anti-aging capability of SBS-modified asphalt remains a critical concern for developing durable pavement materials [8,9,10].

Numerous modification strategies have been proposed to mitigate asphalt aging, including the incorporation of antioxidants, nanomaterials, UV absorbers, composite modifiers, and capsules containing a rejuvenator [11,12,13,14]. Although these approaches can improve certain aging-related properties, many conventional anti-aging additives mainly target either thermo-oxidative aging or UV aging alone, leading to limited comprehensive aging resistance under complex service environments [15,16]. In addition, insufficient compatibility and weak synergistic interactions between modifiers may reduce the long-term effectiveness of composite modification systems [17,18,19,20]. Consequently, the development of multifunctional modifiers capable of simultaneously enhancing rheological performance and comprehensive aging resistance has attracted increasing research interest [21,22].

Zinc diethyldithiocarbamate (ZDC) is a multifunctional organic zinc compound widely used as an antioxidant and stabilizer in polymer materials [23]. Existing studies have shown that ZDC possesses excellent free radical scavenging and peroxide decomposition capabilities, which can effectively suppress oxidation chain reactions during asphalt aging [24,25]. In addition, ZDC has shown great potential for improving fatigue resistance of asphalt binders after aging [26]. Considering the strong viscoelastic enhancement provided by SBS and the antioxidative characteristics of ZDC, the combination of these two modifiers may provide synergistic benefits for improving the durability of asphalt binders.

ZDC presents differentiated interfacial interaction features with the polymeric network and maltene–asphaltene biphasic colloidal system of pristine bitumen, where the bitumen polymeric network constructed via reversible stacking of nanoaggregated asphaltene and surrounding resins largely mediates the oxidative degradation of bitumen matrices [27]. Owing to its inherent amphiphilic structure and interfacial thermodynamic advantages, ZDC preferentially anchors at the maltene–asphaltene interface rather than integrating into the polymeric network through covalent bonding or homogeneous bulk diffusion, with its polar zinc–dithiocarbamate headgroups binding to heteroatom-rich asphaltene via Lewis acid–base interactions and sulfur–metal coordination and its alkyl branches achieving good compatibility with maltene aromatics [26,28]. This stable interfacial anchoring mode enables ZDC to realize multifunctional antioxidative properties, showing a different modification mechanism from conventional bitumen additives with random single-phase dispersion behavior [24].

Superior to traditional metallic dithiocarbamates and zinc antioxidants in chemical and mechanical properties, ZDC can decompose peroxides via non-radical pathways and passivate metals; Ni/Fe dithiocarbamates produce detrimental free radicals through cyclic redox and exhibit pro-oxidant activity, whereas MoDTC combines with arylamines to degrade peroxides and generate tribofilms but is seldom applied in pavements due to insufficient bitumen modification data, high cost and environmental concerns [29]. Zinc dithiocarbamates and structurally distinct ZnDDP deliver favorable antioxidative synergy owing to their identical Zn–S functional groups [30]; their alkyl ligand architecture dominates colloidal dispersion and modification capacity, as short methyl chains may trigger asphaltene precipitation and long butyl chains reduce interfacial adsorption, further impairing antioxidant and rheological protective effects. However, the effects of ZDC on the rheological evolution and aging behavior of SBS-modified asphalt have not yet been investigated, particularly under thermo-oxidative and UV aging conditions [31,32].

**Figure 1:**
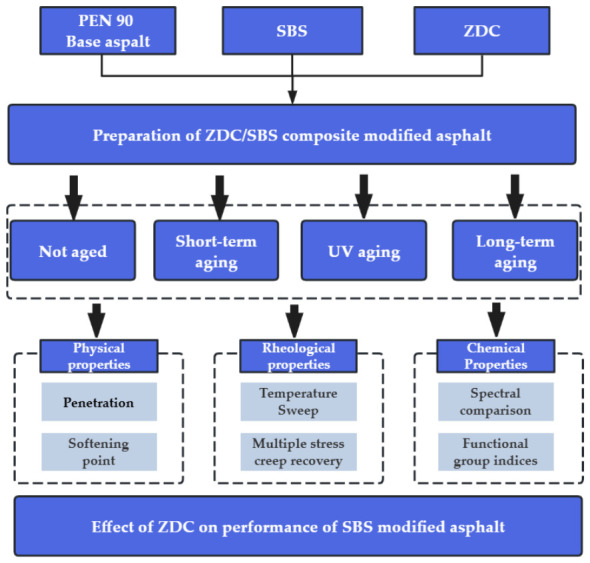
The research methodology of the ZDC/SBS-modified asphalt.

This study aims to evaluate the effects of ZDC on the rheological behavior and aging resistance of SBS-modified asphalt. Conventional physical property tests, temperature sweep tests, multiple stress creep recovery (MSCR) tests, and Fourier transform infrared spectroscopy (FTIR) were conducted to investigate the physical properties, rheological performance, and chemical evolution of the modified binders before and after aging. The influence of ZDC on deformation resistance, elastic recovery behavior, and oxidation inhibition mechanisms was studied. The research methodology is shown in Figure 1.

## 2. Materials and Methods

### 2.1. Materials

This study utilized PEN 90 base asphalt produced in Macheng, Hubei Province, to prepare the SBS-modified asphalt first before preparing different aged binders, and the properties of the asphalt can be found in our previous study [33]. The SBS particles were supplied by Dushanzi Petrochemical Company (Korla, China). The SBS content was 4.5%, and the basic physical properties are presented in Table 1.

ZDC (zinc diethyldithiocarbamate) powder was supplied by Macklin (Shanghai, China). The molecular formula of ZDC is C_10_H_20_N_2_S_4_Zn, which has a purity of ≥97.5%, a melting point of 172 °C, a density of 1.48 g/cm^3^, and exhibits favorable compatibility with the PEN 90 base asphalt. Three gradient dosages with 1%, 3%, and 5% were selected to cover the typical applicable concentration range of asphalt dithiocarbamate modifiers and systematically characterize the dosage-dependent anti-aging performance of ZDC [26,28].

### 2.2. Asphalt Sample Preparation and Aging Protocols

#### 2.2.1. Asphalt Sample Preparation

**Figure 2:**
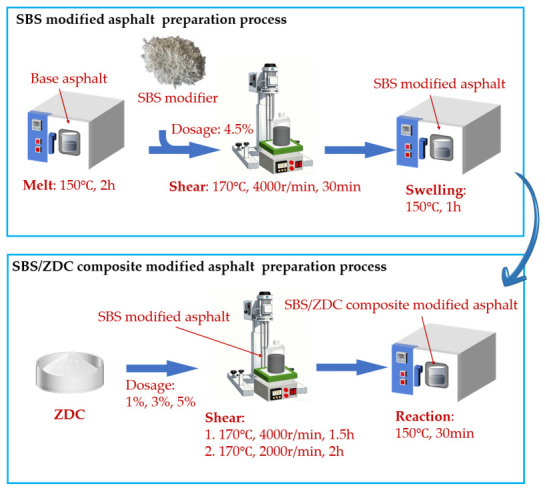
Preparation process for SBS and ZDC/SBS-modified asphalt samples.

SBS-modified asphalt was prepared using the melt blending method [38]. The preparation process for the asphalt samples is illustrated in Figure 2. The preparation process was as follows: A specific mass of base asphalt was added to the modification tank, which was then heated to 150 °C and maintained for 2 h. Then, 4.5% SBS modifier was mixed and sheared at 170 °C and 4000 r/min for 30 min. After high-speed shearing, the mixture was kept at 150 °C for 1 h to complete the swelling of SBS, obtaining SBS-modified asphalt.

Subsequently, ZDC powder (with dosages of 1%, 3%, and 5%) was added to the prepared SBS-modified asphalt, and the mixture was subjected to two-stage shearing; first at 170 °C and 4000 r/min for one and a half hours, then at 170 °C and 2000 r/min for 2 h. Finally, the SBS/ZDC-modified asphalt was prepared after curing at 150 °C for 30 min.

#### 2.2.2. Asphalt Aging Protocols

The short-term aging test for asphalt samples was conducted in accordance with ASTM D2872 [39] using a rotating film oven (RTFO). The key test parameters are set as follows: aging temperature 163 °C, rack rotation speed 15 r/min, hot air flow rate 4.0 L/min, and continuous aging duration of 5 h. This RTFO test simulates short-term thermo-oxidative aging during the asphalt mixing, storage, transportation, and paving stages, providing aged specimens for subsequent performance testing.

The UV aging test was conducted following ASTM D4799 [40] which simulated the aging from outdoor sunlight during the long-term service. Short-term aged asphalt samples were subjected to the UV light in a SC/ZN-P UV-aging chamber. The specimens were exposed for 120 h under conditions of intense UVA-340 UV light (0.68 W/m^2^ at 340 nm), 60 ± 3 °C, and a rotation rate of 3–5 r/min.

**Figure 3:**
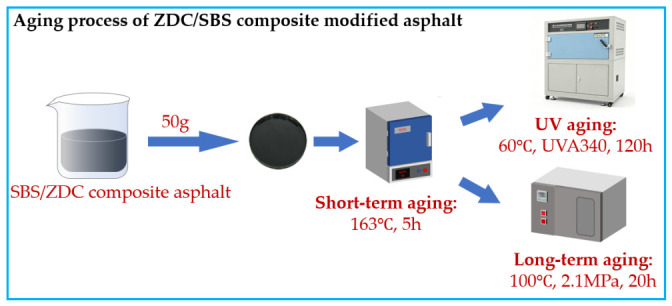
Aging process of ZDC/SBS-modified asphalt.

The long-term aging test was conducted following ASTM D6521 [41] using a Pressure Aging Vessel PAV-1 (Beijing Samyon Instruments Co., Ltd., Beijing, China). This method involves subjecting asphalt specimens that have undergone short-term aging to 20 h of pressurized heating under high-temperature and high-pressure conditions, simulating the actual degree of aging of asphalt pavements after 5 to 10 years of service. The PAV test is performed at 100 °C and 2.1 MPa atmospheric pressure. Figure 3 shows aging process of ZDC/SBS-modified asphalt.

### 2.3. Physical Property Tests

The penetration and softening point of asphalt were investigated following ASTM D5 [42] and ASTM D36 [43], respectively. A higher penetration value indicates that the asphalt is softer and has lower viscosity. The softening point indicates the temperature at which asphalt begins to soften from a solid or semi-solid state and is a key indicator of its high-temperature stability and resistance to rutting.

### 2.4. Rheological Properties Tests

#### 2.4.1. Temperature Sweep (TS)

Following the AASHTO T315 standard [44], parallel-plate specimens with a thickness of 1 mm and a diameter of 25 mm were selected for TS testing. During the test, the angular frequency was fixed at 10 rad/s, the target strain was set at 12%, and the temperature control range was 30–80 °C. An Anton Paar MCR102 Dynamic Shear Rheometer (DSR, Graz, Austria) was used to test the dynamic rheological properties of the asphalt. The complex shear modulus (|G*|) and phase angle (δ) of the asphalt specimens were measured to characterize the evolution of the asphalt’s viscoelastic properties during the aging process [45].

#### 2.4.2. Multiple Stress Creep Recovery (MSCR) Test

In accordance with the AASHTO T350 [46] test specification, multiple stress creep recovery tests were conducted to determine the asphalt’s creep recovery rate (*R*) and irreversible creep modulus ($J_{nr}$), thereby evaluating the asphalt’s high-temperature deformation resistance and elastic recovery characteristics. The test temperature was set at 64 °C. A 25 mm parallel-plate fixture was used with a 1 mm gap between the specimens. Tests were conducted under two stress levels: 0.1 kPa and 3.2 kPa. Each loading cycle lasted 1 s, followed by a 9 s unloading and recovery period. The test consisted of 10 consecutive cycles. $J_{nr}$ is defined as the ratio of irreversible strain to applied stress in the MSCR test, while *R* is used to characterize the elastic response of the binder and its stress dependence. Both are core indicators for evaluating asphalt’s high-temperature rutting resistance and stress sensitivity. $J_{nr}$ and *R* are calculated using the following formulas:

(1) $$J_{nr}=\frac{\gamma_{nr}-\gamma_{0}}{\tau }$$

(2) $$R=\frac{\gamma_{p}-\gamma_{nr}}{\gamma_{p}-\gamma_{0}}$$

where:

*γ*_p_ is the peak strain for each creep recovery cycle;

*γ*_nr_ is the residual strain for each creep recovery cycle;

*γ*_0_ is the initial strain for each creep recovery cycle.

### 2.5. Chemical Performance Testing

Infrared spectroscopy is an analytical method that identifies the molecular structure of a substance by analyzing its absorption of infrared light [47,48]. It can determine functional groups based on the position and intensity of characteristic absorption peaks or perform qualitative identification by comparing the results with standard spectral libraries. This method allows for a clear observation and assessment of the chemical effects of adding ZDC to SBS-modified asphalt, providing valuable insights into the chemical behavior of ZDC in the context of subsequent short-term aging, long-term aging, and UV aging. The IR spectroscopy experiments in this study were conducted using a Perkin Elmer Spectrum 100 FTIR spectrometer (PerkinElmer, Waltham, MA, USA).

Asphalt aging implies the occurrence of oxidation reactions, primarily manifested by a significant increase in the content of carbonyl (C=O) and sulfoxide (S=O) functional groups. Since the subject of this study is SBS-modified asphalt, the carbonyl (C=O), sulfoxide (S=O), and SBS characteristic peaks at 1696 cm^−1^, 1030 cm^−1^, and 966 cm^−1^ in the infrared spectrum were selected as key analytical indicators. The areas of these characteristic peaks were calculatedto compute the carbonyl index (*I_C_*), the sulfoxide index (*I_S_*), and the SBS index (*I_SBS_*), we quantitatively analyze the aging effects on the chemical composition and molecular structure of asphalt. All asphalt samples were tested by scanning 32 times in the wavenumber range of 500–4000 cm^−1^ at a resolution of 4 cm^−1^.

(3) $$I_{C}=\frac{A_{1696}}{\sum A}$$

(4) $$I_{S}=\frac{A_{1030}}{\sum A}$$

(5) $$I_{SBS}=\frac{A_{966}}{\sum A}$$

(6) $$\sum A=A_{725}+A_{746}+A_{810}+A_{865}+A_{966}+A_{1030}+A_{1373}+A_{1456}+A_{1601}+A_{1696}+A_{2853}+A_{2921}$$

where:

*I_C_* is the carbonyl index;

$I_{S}$ is the sulfoxide index;

$I_{SBS}$ is the Butadiene Index;

∑A is the sum of the peak areas of all reference characteristic peaks;

A*_i_* is the peak area at the corresponding wave number of the characteristic functional group.

## 3. Results and Discussion

### 3.1. Physical Properties

**Figure 4:**
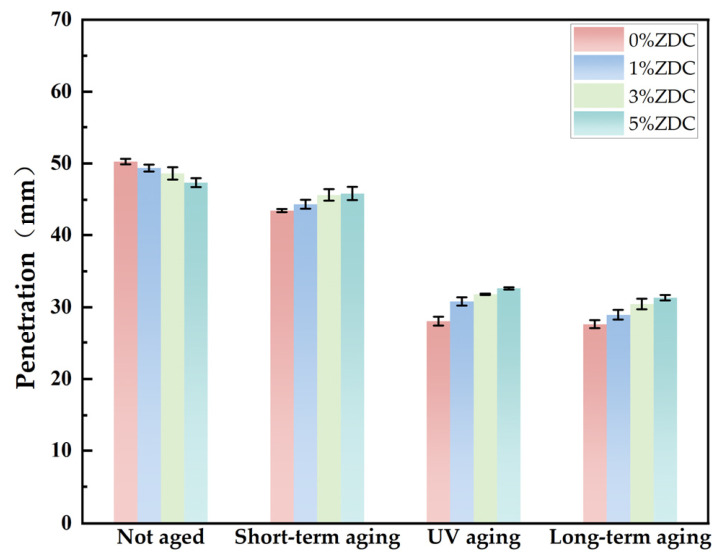
Penetration of ZDC/SBS-modified asphalt.

Figure 4 shows the influence of three ZDC dosages on the penetration of asphalt at 25 °C. The penetration of all asphalt samples gradually declines during the aging process. The penetration of neat SBS-modified asphalt decreases significantly from 50.3 (0.1 mm) to 27.6 (0.1 mm), which leads to the asphalt binder exhibiting higher hardness and brittleness. The addition of ZDC can effectively suppress the aging deterioration of SBS-modified asphalt and improve its penetration retention property. Under the same aging conditions, the penetration retention effect is enhanced with the increase in ZDC dosages to 1%, 3% and 5%. In addition, no significant difference in penetration value is found when the ZDC dosage rises from 3% to 5%.

It is noticed that ZDC showed a higher anti-aging improvement during the UV aging process compared to long-term aging, which is demonstrated by the penetration difference before and after the aging test; e.g., the penetration difference for SBS-modified asphalt incorporated with 5% ZDC is 4.56 (0.1 mm) for UV aging, whereas this value is 3.67 (0.1 mm) for long-term aging. This observation is consistent with the UV-protective mechanisms reported in prior studies, where ZDC may contribute to UV aging resistance by scavenging UV-induced free radicals, decomposing peroxides, and inducing UV scattering via zinc-containing components, thereby potentially interrupting UV-triggered oxidative chain degradation [49,50].

**Figure 5:**
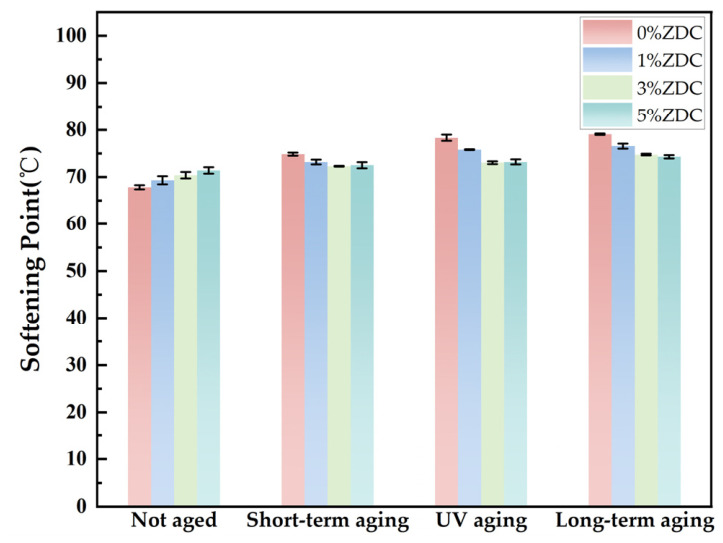
Softening point of ZDC/SBS-modified asphalt.

Figure 5 shows the effect of various ZDC dosages on the softening point of asphalt. During aging, the softening point of neat SBS-modified asphalt increases from 67.8 °C to 79.1 °C. This phenomenon reveals that aging increases the high-temperature stiffness of the asphalt, reduces its cohesion, and amplifies temperature susceptibility. Incorporating ZDC can effectively reduce the softening point of aged SBS-modified asphalt, and the softening point exhibits a negative correlation with ZDC dosage, which shows a similar trend to the penetration results. ZDC can soften the asphalt matrix and inhibit the aging of asphalt binders. It was also found that 3% and 5% ZDC dosages achieved the optimum anti-aging performance among all SBS-modified asphalt samples. The softening point difference between SBS-modified asphalt with 5% ZDC and SBS-modified asphalt is 5.1 °C after UV aging, and this gap slightly rises to 5.2 °C after long-term aging. It means that the incorporation of ZDC has a similar anti-aging effect under UV aging and long-term thermo-oxidative aging.

### 3.2. Rheological Properties

#### 3.2.1. Temperature Sweep Analysis

**Figure 6:**
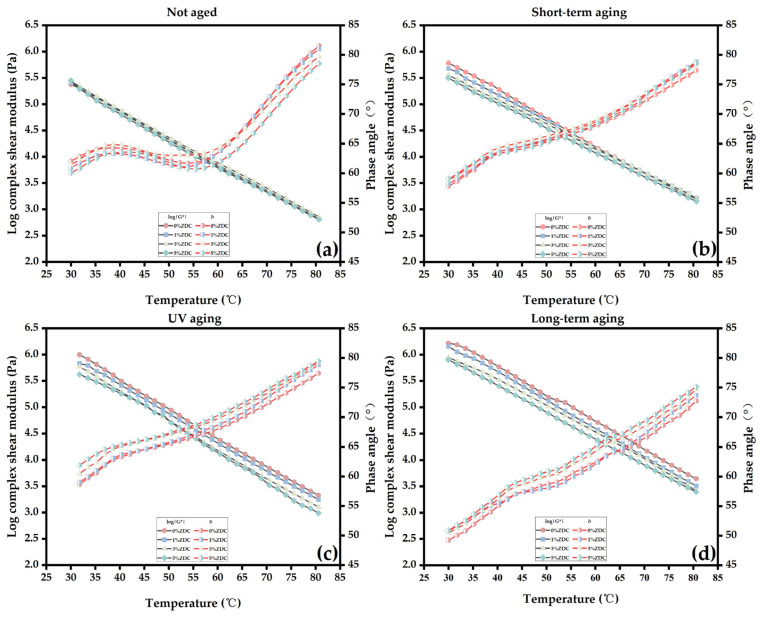
Complex shear modulus and phase angle for unaged asphalt and aged asphalt: (**a**) the complex shear modulus and phase angle for unaged asphalt, (**b**) the complex shear modulus and phase angle for short-term aged asphalt, (**c**) the complex shear modulus and phase angle for UV aging, and (**d**) the complex shear modulus and phase angle for long-term aged asphalt.

Figure 6 shows the complex shear modulus and phase angle results of SBS-modified asphalt samples with different ZDC dosages under various aging conditions. Specifically, lg|G*| represents the shear deformation resistance capacity of asphalt, while the phase angle (δ) describes the mechanical response lag between its viscous and elastic fractions. For all aged asphalt specimens, lg|G*| continued to decrease as the test temperature increased, while δ showed an increasing trend. In unaged asphalt, lg|G*| decreases significantly with rising temperature. The phase angle results rise slowly at first, then decreased and followed by a sharp increase.

It was also found that ZDC can mitigate the changes in the complex shear modulus and phase angle of asphalt samples during the aging process. With the increase in ZDC dosage, the phase angle (δ) of aged asphalt decreased gradually and an optimum performance was observed at the ZDC dosage of 5%. The temperature sweep results indicate that the addition of ZDC can effectively inhibit the degradation of rheological properties caused by long-term thermo-oxidative aging and ultraviolet aging. Consistent with the penetration results, the amplitude difference in lg|G*| curves between 5% ZDC-modified asphalt and neat SBS-modified asphalt was more prominent under UV aging than under long-term thermo-oxidative aging.

#### 3.2.2. Multiple Stress Creep Recovery Analysis

**Figure 7:**
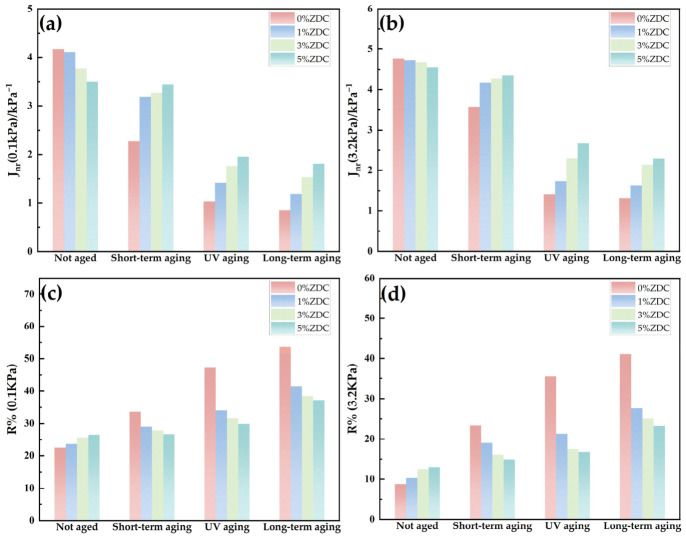
MSCR indicators of ZDC/SBS-modified asphalt: (**a**) the non-recoverable creep compliance under 0.1 kPa, (**b**) the non-recoverable creep compliance under 3.2 kPa, (**c**) the creep recovery rate under 0.1 kPa and (**d**) the creep recovery rate under 3.2 kPa.

Figure 7 presents the irreversible creep compliance ($J_{nr}$) and creep recovery rate (*R*) of SBS-modified asphalt samples with different ZDC dosages under various aging conditions at shear stresses of 0.1 kPa and 3.2 kPa, respectively. At the same ZDC dosage, the irreversible creep compliance ($J_{nr}$) decreases markedly with the aggravation of aging degree, whereas *R* exhibits a continuous increasing trend for all asphalt specimens. Specifically, asphalt without ZDC modification is the most susceptible to aging-induced performance deterioration, which shows more pronounced variations in $J_{nr}$ and *R*.

When the applied shear stress rises from 0.1 kPa to 3.2 kPa, the $J_{nr}$ values of all asphalt samples increase substantially, while the *R* declines universally. This indicates that elevated stress loading impairs the elastic resilience capacity of asphalt materials. Under identical aging conditions and shear stress levels, the incorporation of ZDC is capable of alleviating excessive hardening behavior of aged asphalt and effectively promoting creep recovery performance. With the increase in ZDC dosage, the stress sensitivity of modified asphalt is gradually weakened, and its high-temperature permanent deformation resistance as well as creep relaxation characteristics are remarkably enhanced. Regardless of applied load levels, the difference in $J_{nr}$ between ZDC/SBS-modified asphalt and conventional SBS-modified asphalt under UV aging is larger than that under long-term aging. Similarly, the difference in *R* between the two asphalt binders under UV aging is smaller than that under long-term aging. This is consistent with the enhanced UV aging resistance of ZDC composite SBS-modified asphalt observed in physical property tests.

### 3.3. Chemical Properties

#### 3.3.1. Spectral Comparison Analysis

The observed suppression of oxidative functional groups suggests that ZDC may exert dual anti-oxidative effects to interrupt asphalt autoxidation chains, as proposed in the previous literature; its dithiocarbamate groups may scavenge peroxyl radicals for chain termination, while the central Zn^2+^ may catalytically decompose hydroperoxides through non-radical pathways [51]. Such synergistic radical suppression, as documented in earlier work, may inhibit autocatalytic oxidation and retard the oxidative degradation of the asphalt binder and SBS modifier [52].

**Figure 8:**
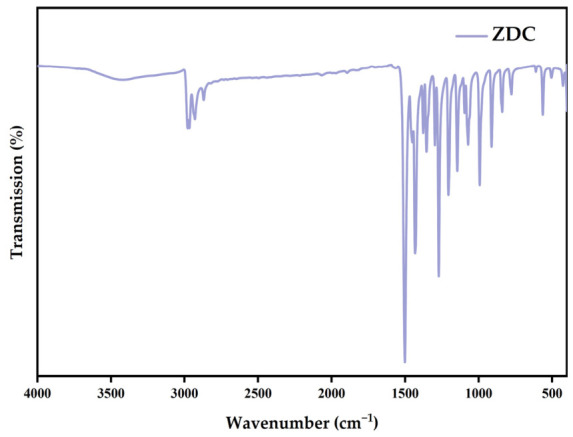
FTIR spectrum of ZDC.

Figure 8 presents the FTIR spectrum of the ZDC modifier. The characteristic absorption peak assigned to the alkyl C–H stretching vibration is observed at 2900~3000 cm^−1^, which corresponds to the ethyl side chain in the molecular structure of ZDC. The intense absorption peak at 1400~1500 cm^−1^ originates from the stretching vibration of the C–N bond in the dithioaminocarbonate group. Meanwhile, the series of strong characteristic peaks in the range of 1000~250 cm^−1^ are attributed to the vibrational absorption of C=S and C–S bonds, which serve as the characteristic signature peaks of dithioaminocarbonate compounds.

**Figure 9:**
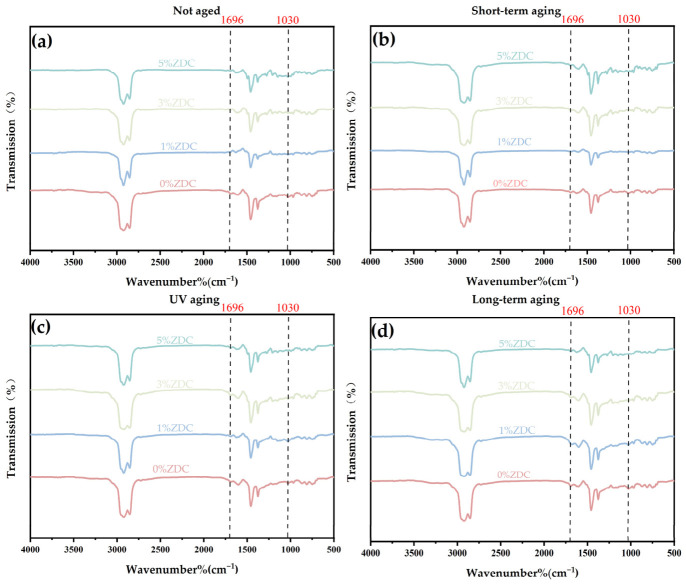
FTIR spectra of ZDC/SBS-modified asphalt: (**a**) the FTIR spectrum for unaged asphalt, (**b**) the FTIR spectrum for short-term aged asphalt, (**c**) the FTIR spectrum for UV aged asphalt and (**d**) the FTIR spectrum for long-term aged asphalt.

It is generally accepted that thermo-oxidation and photo-oxidation induce SBS degradation via chain scission and over-crosslinking. The preserved SBS index in ZDC-modified samples suggests that ZDC may help retain the integrity of the SBS triblock structure and asphalt network elasticity, likely by eliminating peroxyl radicals and hydroperoxides as reported in previous studies [26,28]. Figure 9 presents the FTIR spectra of SBS-modified asphalt with different ZDC dosages under various aging conditions. The main characteristic absorption peaks of all asphalt specimens remain highly consistent, indicating that the incorporation of ZDC does not alter the fundamental chemical structure of asphalt. No new characteristic peaks are detected after ZDC modification, suggesting that the interaction between ZDC and asphalt is mainly physical blending rather than chemical reaction.

As the aging degree increases, the intensities of the 1696 cm^−1^ and 1030 cm^−1^ peaks gradually increase, indicating that thermo-oxidative reactions continuously occur during aging, leading to the formation of more polar oxygen-containing functional groups and the aggravation of asphalt aging (for both long-term aging and UV aging).

Figure 9 further indicates that, under the same aging conditions, the carbonyl and sulfoxide peak intensities gradually decline with increasing ZDC dosage. This demonstrates that ZDC can effectively inhibit the generation of oxidative functional groups during aging and mitigate the thermal-oxidative aging of asphalt. The reduction in oxidation-related peaks may be attributed to the antioxidant effect of ZDC, which can block free radical chain reactions and slow down the oxidative degradation of the asphalt matrix.

#### 3.3.2. Analysis of Functional Group Indices

**Figure 10:**
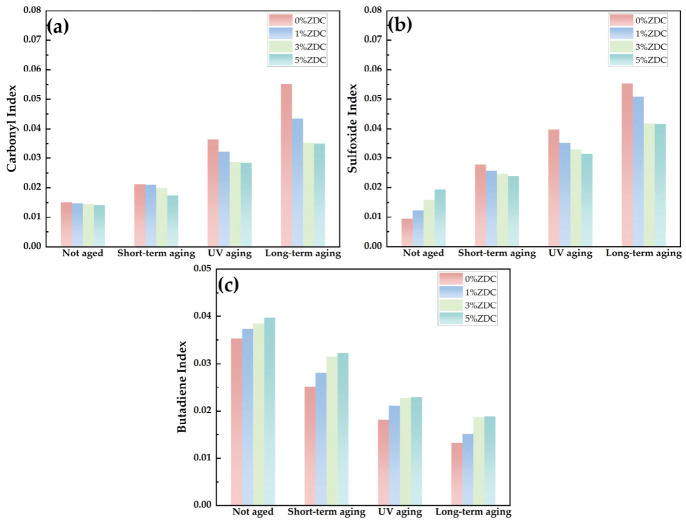
Functional group indices of ZDC/SBS-modified asphalt under different aging conditions: (**a**) Carbonyl Index, (**b**) Sulfoxide Index and (**c**) Butadiene Index.

Figure 10 shows the carbonyl index (CI), sulfoxide index (SI), and SBS structural index of SBS-modified asphalt samples with different ZDC dosages under various aging conditions. It shows that, with the increase in aging degree, the CI and SI values of all asphalt samples gradually increase, while the SBS index gradually decreases, indicating that oxidative aging reactions continuously occur and the SBS structure is progressively degraded during the aging process.

As shown in Figure 10a,b, under the same aging condition, the CI and SI values gradually decrease with the increase in ZDC dosage from 0% to 5%, implying that ZDC can effectively inhibit the formation of oxidation-related functional groups during aging. In the non-aged condition, the SI increases slightly while the CI decreases slightly, which may be attributed to the spectral overlap between the characteristic sulfur-containing peaks of ZDC and the intrinsic sulfoxide absorption bands of asphalt.

Figure 10c shows that the SBS index of all asphalt samples decrease after aging, while the SBS index gradually increases with increasing ZDC dosage under the same aging condition. Among them, the SBS index of the 0% ZDC asphalt decreases most significantly after long-term aging, whereas the 3% and 5% ZDC samples maintain relatively high SBS indices throughout the aging process, indicating that ZDC can effectively retard the degradation of the SBS structure during aging. Furthermore, the protective effect becomes more pronounced with increasing ZDC dosage, which is consistent with the variation trends of CI and SI. Compared with an unmodified SBS asphalt binder, the addition of 5% ZDC reduced CI and SI by 44.77% and 60.40% after long-term aging, respectively, and such inhibition efficiency appears to be favorable compared with that of conventional phenolic and amine antioxidants reported in the relevant literature [26,27].

In summary, the FTIR characterization results verify that ZDC can effectively retard the aging process of asphalt by inhibiting the generation of carbonyl and sulfoxide functional groups. ZDC performs a prominent inhibitory effect on both long-term thermo-oxidative aging and UV aging, and can maintain the integrity of SBS molecular structure, thereby improving the aging resistance and long-term service stability of modified aphalt.

Rheological characterization confirms an evident micro–macro aging linkage: the elevated carbonyl index coincides with enhanced complex shear modulus and declined non-recoverable creep compliance $J_{nr}$. ZDC provides stronger anti-ultraviolet aging performance than its anti-thermo-oxidation capacity. After aging, ZDC-free specimens present a three times higher increment of the carbonyl index and a far more severe rheological property degradation. Regarding SBS-modified asphalt incorporated with 5% ZDC, the improvement rates of $J_{nr}$ and *R* reach 41.54% and 80.56% under UV aging, which are higher than the corresponding values of 31.54% and 78.32% obtained under long-term thermo-oxidative aging.

Experimental data preliminarily verify that ZDC improves the anti-oxidation and rheological performance of SBS-modified asphalt, yet its large-scale engineering promotion requires systematic evaluation of industrial compatibility, field performance and environmental safety. Further studies on mixing manufacturability, long-term performance of virgin asphalt mixtures and zinc leaching risks are necessary to support its practical application.

## 4. Conclusions

This study investigated the effects of zinc diethyldithiocarbamate (ZDC) on the rheological properties and aging resistance of SBS-modified asphalt through physical property tests, rheological characterization, and FTIR analysis. The following conclusions can be drawn:

1. Penetration and softening point tests prove aging hardens SBS-modified asphalt, and long-term aging brings more severe performance loss than UV aging. Compared with aged neat SBS asphalt, ZDC mitigates aging deterioration, boosting penetration retention by 21.78% and restraining softening point growth by 78.45%. The anti-aging capacity elevates with ZDC dosage, and 3% and 5% ZDC deliver the best anti-aging performance.
2. DSR temperature sweep tests demonstrated that aging remarkably altered the complex shear modulus and phase angle of asphalt samples. The incorporation of ZDC effectively restrained the aging-induced variations in rheological parameters, mitigated asphalt stiffening, and retained favorable viscous-elastic characteristics. Increasing ZDC dosage gradually reduced the phase angle and enhanced high-temperature deformation resistance.
3. MSCR results indicate long-term thermal aging causes severer asphalt performance deterioration than UV exposure, reducing deformability and aggravating permanent deformation. Neat SBS-modified asphalt exhibits severe aging hardening, with a 72.53% drop in $J_{nr}$ and tripled R value. Incorporating ZDC alleviates aging hardening and enhances elastic recovery and rutting resistance; higher ZDC dosages further reduce stress sensitivity and improve creep resistance. After UV aging, the asphalt binder with 5% ZDC achieves 41.54% and 80.56% improvements in $J_{nr}$ and R.
4. FTIR spectra demonstrate that both long-term thermo-oxidative and UV aging promote carbonyl and sulfoxide generation and degrade SBS chains. Thermo-oxidative aging induces more severe chemical deterioration, raising the carbonyl index to three times its original value and lowering the SBS index by 62.52%. ZDC suppresses oxidative reactions and limits oxygenated group accumulation; 5% ZDC cuts carbonyl growth by 44.77% under long-term aging and markedly delays SBS double-bond cleavage across all aging environments.
5. ZDC exhibits stronger anti-aging performance against UV aging compared with thermo-oxidative aging, which is demonstrated in the penetration and rheological test results. As such, ZDC can improve the comprehensive aging resistance of polymer-modified asphalt binders, which makes it a promising functional modifier for asphalt pavement materials under complex environmental aging conditions.

Although ZDC modification improves the rheological properties and thermo-oxidative aging resistance of SBS-modified asphalt, this study lacks a systematic assessment of its low-temperature performance, particularly the BBR-based low-temperature cracking resistance. Future studies may explore the tank storage stability and density-induced particle sedimentation of ZDC-modified asphalt, and further evaluate its production cost, industrial feasibility, long-term field performance and environmental impacts to promote the practical application of ZDC-modified asphalt binders.

## Figures and Tables

**Table 1:** Physical properties of SBS.

Performance Item	Unit	Test Condition	Measured	Standard
Melt Flow Rate	g/10 min	190 °C/5.0 kg	18	GB/T 3682.1-2018 [34]
Density	g/cm^3^	23 °C	0.958	GB/T 1033.1-2008 [35]
Elongation	%	23 °C, 50 mm/min	40	GB/T 1040.2-2022 [36]
Tensile Strength	MPa	23 °C, 50 mm/min	27.5	GB/T 1040.2-2022 [36]
Ash Content	%	850 °C	0.03	GB/T 9345.1-2008 [37]

## Data Availability

The original contributions presented in this study are included in the article. Further inquiries can be directed to the corresponding author.
